# Educational Attainment and Women’s Environmental Mastery in Midlife: Findings From a British Birth Cohort Study

**DOI:** 10.1177/0091415016641687

**Published:** 2016-04-05

**Authors:** Mai Stafford, Dorly Deeg, Diana Kuh

**Affiliations:** 1 MRC Unit for Lifelong Health and Ageing at UCL, London, UK; 2 VU University Medical Center, EMGO Institute—LASA, Amsterdam, the Netherlands

**Keywords:** life course, prospective, self-efficacy, control, mental well-being

## Abstract

Using data from 1,184 women in the MRC National Survey of Health and Development, we estimated associations between education and Ryff’s environmental mastery scale scores at age 52. Confirmatory factor analysis indicated two subscales, here termed mastery skills and mastery accomplishments. Low education was associated with higher mastery skills. This was partly explained by childhood socioeconomic position, as mastery was lower among those with fathers in the most and least advantaged occupational classes. Education was not associated with mastery accomplishments in unadjusted models. Lower ambitions for family/home were associated with higher mastery accomplishments and may have partly suppressed as an association between education and mastery accomplishments. This study highlights childhood as well as adult correlates of mastery and adds to mounting evidence that higher mastery is not universally found among those who are more educated.

Mastery is seen as being an integral component of ageing well (Rowe & Kahn, 1997; Ryff, 1989). In older age, having a greater sense of mastery, that is a stronger belief that one can control or influence one’s environment and outcomes, may be protective for mortality, including cardiovascular disease and all-cause mortality (Bosma, Schrivers, & Mackenbach, 1999; Menec & Chipperfield, 1997; Surtees et al., 2010) and institutionalization (Cooper, Huisman, Kuh, & Deeg, 2011). Longitudinal analysis suggests that, on average, mastery increases from young adulthood to midlife and subsequently declines (Krause, 2007; Mirowsky & Ross, 2007; Schieman, 2001). The sixth decade may be the peak for levels of mastery. A sense of mastery is thought to be accumulated over the life course and based on integration of previous and current experiences (Mirowsky & Ross, 2007; Pearlin, Nguyen, Schieman, & Milkie, 2007). Here, we focus on describing the relationship between education and mastery in a cohort of women born in Britain in the 1940s. Although a well-established literature has documented a positive association between educational attainment and mastery, we submit that this association may not be universally found but may be contingent on context and culture.

## Measurement of Mastery

The process of psychological development through the life course includes development of the ability to manipulate and master one’s environment. This includes managing complex environments. It also includes the ability to select, shape, and achieve circumstances and environments that relate well to one’s goals and values. Though there are alternative mastery scales, the focus of the current study is on environmental mastery as conceived by Ryff (1989). This scale was developed to capture the extent to which a person is ageing well and was included in the survey on which the current analyses were based. It is one of the six scales designed by Ryff to capture positive psychological functioning and several studies have examined their psychometric properties (Abbott et al., 2006; Ryff ,1989; Springer & Hauser, 2006) though none have focused on the mastery scale alone. It is plausible that the experiences and circumstances that contribute to the development of skills to manage complex environments and multiple roles differ from those that enable people to choose and achieve circumstances that align with their goals. This might suggest the existence of subscales within the overall environmental mastery scale.

The construct of mastery has considerable overlap with that of sense of control. Some have noted that mastery refers to management of the particular circumstances of the individual’s life rather than having a more generalized sense of personal control (Pearlin et al., 2007). Environmental mastery emphasizes the ability to choose or change the surrounding context using physical or mental actions as well as being able to control events (Ryff, 1989). Although there may be some conceptual differences, the current study builds on empirical evidence from studies investigating the determinants of both mastery and perceived control.

## Pathways Linking Education and Mastery

Education is considered a context for developing mastery as it provides the opportunity to develop skills and psychological characteristics that promote mastery, such as experiencing success and confidence in learning, and developing problem solving, planning, and communication skills (Mirowsky & Ross, 2007). Status attainment is another mechanism by which education is conceptually linked to mastery (Pearlin et al., 1997). Greater educational attainment is one indicator of a person’s social status within society and this success in itself may promote the belief that a person can master their circumstances. Education also indicates the possession of skills that enhance a person’s position in the labor market and attract financial rewards that continue to signal higher social status throughout adulthood. In many societies, these also translate into tangible benefits and accomplishments and reduce the likelihood of experiencing stressors that may reduce mastery.

These benefits include higher income, occupational status, and autonomy at work (Singh-Manoux, Clarke, & Marmot, 2002). Education thus increases access to social and economic resources, such as income to purchase goods and services, work characterized by high levels of autonomy, and leadership opportunities in the workplace and elsewhere, that facilitate being able to control one’s environment and are positively associated with mastery (Cassidy & Davies, 2003; Kaplan, Shema, & Leite, 2008; Pearlin et al., 2007; Wolinsky, Wyrwich, Babu, Kroenke, & Tierney, 2003).

On the other hand, education may reduce the likelihood of experiencing stressors that have been associated with poorer sense of mastery. Greater educational attainment is associated with lower likelihood of family-related life events including marital problems and having problems with one’s children (Harkonen & Dronkers, 2006), which in turn have been related to diminished mastery or control (Cassidy & Davies, 2003; Krause, 1993; Pearlin & Schooler, 1978; Pearlin et al., 2007; Schieman & Turner, 1998; Wolinsky et al., 2003). Greater educational attainment may also reduce the exposure to physical and mental ill health (Baltes, Wahl, & Schmid-Furstoss, 1990; Cooper et al., 2011; Infurna, Gerstorf, Ram, Schupp, & Wagner, 2011; Pearlin et al., 2007; Wolinsky et al., 2003) which challenges a person’s sense of mastery or control.

## Existing Evidence on the Mastery–Education Relationship

Several studies document a positive and dose–response relationship between education and mastery (Cassidy & Davis, 2003; Dalgard, Mykletun, Rognerud, Johansen, & Zahl, 2007; Pearlin, Lieberman, Menaghan, & Mullan, 1981) or sense of control (Mirowsky, 1995; Mirowsky & Ross, 2007; Schieman, 2001; Specht, Egloff, & Schmukle, 2013) in all-age adults and in middle- and older-aged adults (Kubzansky, Berkman, Glass, & Seeman, 1998; Pearlin et al., 2007; Slagsvold & Sorensen, 2008). Although the current literature is consistent, most studies estimated the association between education and mastery averaged across men and women. Studies of gender differences in the impact of education on mastery are few in number but one found education to be positively associated with mastery among women but not among men (Cassidy & Davies, 2003). Furthermore, the literature is heavily weighted toward U.S. samples. To date, one UK study of people born in 1970 has shown that education is positively associated with internal locus of control at age 30 (Flouri & Hawkes, 2008). Investigations of mastery in older UK cohorts may not show the same pattern. Compared with the United States, the United Kingdom had historically lower educational attainment among people born in the early 1940s and earlier (Gurria, 2011). UK women born at this time were paid substantially lower wages than their male counterparts when they entered the labor market, a difference which has reduced though not disappeared in more recent cohorts (Joshi & Paci, 1998). The majority had become mothers by their mid 20s, even among the most highly educated group, and they had a longer employment break after the birth of their first child compared with more recent cohorts (Macran, Joshi, & Dex, 1996). Thus, there may be international and cohort differences in the tangible benefits arising from education that may modify the education–mastery link.

## Complexities in the Education–Mastery Relationship

Despite the body of evidence supporting a positive association between education and mastery, and plausible explanatory pathways, some recent studies have suggested that the association may not be straightforward. Difficult experiences, such as social disadvantage, may require people to become more self-reliant and to develop skills to cope with these negative life events and experiences. In Ryff and colleagues’ study of ethnic minority status and well-being, African American and Mexican American people in the United States were found to have higher mastery than Caucasians (Ryff, Crey, & Hughes, 2003). These findings are in line with the notion of steeling (Rutter, 2006), whereby earlier circumstances or experiences which are challenging but not overwhelming, in contexts which provide resources to meet those challenges, should facilitate the accumulation of mastery (Luthar, Cicchetti, & Becker, 2000; Pearlin et al., 2007) and with the biological concept of hormesis (Calabrese, Dhawan, Kapoor, Iavicoli, & Calabrese, 2015) which denotes adaptability to low-dose environmental challenges and enhances survival. Based on this, we hypothesize that moderate levels of childhood socioeconomic disadvantage will be associated with the highest levels of mastery and will negatively confound (or suppress) the association between education and mastery.

An additional consideration is that a reported high sense of mastery may be achieved through two processes (Rothbaum, Weisz, & Snyder, 1982). High mastery may reflect primary control, that is, directly controlling behavior to alter one’s environment or roles in line with one’s wishes. It may alternatively reflect secondary control, which is achieved through reducing the scope of one’s goals and values to bring them in line with the environment. For example, one response to chronic failure to attain a goal may be to disengage from that goal and possibly engage with a more apparently achievable one (Heckhausen, Wrosch, & Schulz, 2010; Wrosch, Scheier, Miller, Schulz, & Carver, 2003). It has been proposed that early disadvantage may lead to decreased motivation for achievement and the construction of a more limited set of goals (McLanahan, 1985). We hypothesize that goals in two key life domains—work and family—will be more limited among those with less compared with more education. We further hypothesize that a more limited set of goals will be associated with greater reported mastery in relation to one’s circumstances and achievements.

## Study Aims

**Figure 1.**
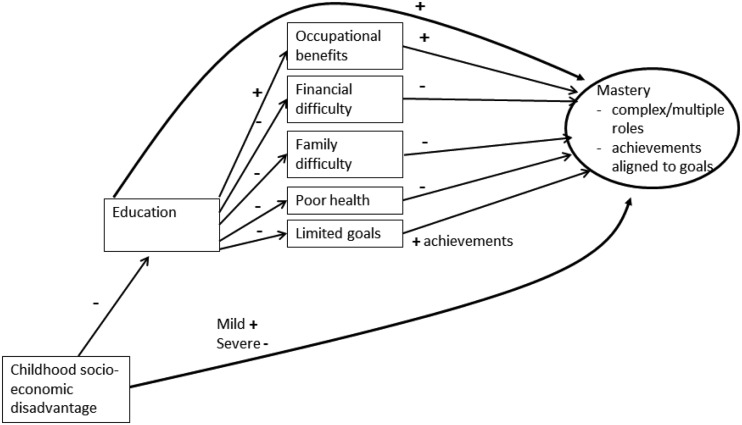
Conceptual model linking education to midlife mastery.

Using prospective data from a cohort study of people born in England, Scotland, or Wales in 1946, we set out to describe the association between educational attainment and women’s mastery in midlife and examine the contributions of tangible benefits in the occupational, financial, family, and health domains to this association. Based on the previous literature, we expected to find greater mastery among more educated women. However, we also considered the possibility that a positive association between education and mastery may be suppressed by the experience of early social disadvantage or by setting more limited goals for one’s life or may not be evident in this sample because of the more limited occupational opportunities available to highly educated women born in the United Kingdom in 1946. The hypothesized associations are summarized in Figure 1. Here, we considered only women because midlife mastery data were not available for men in this cohort.

## Data and Methods

### Study Design

The MRC National Survey of Health and Development (NSHD) is a socially stratified sample of 5,362 singleton births (2,547 women) from 1 week of March 1946 in Britain, with regular follow-up across life. Between the ages of 47 and 54, an annual sub-study of women’s health was conducted on 1,778 (70%) of the original cohort of women. Women who had died (6%), had previously withdrawn from the study (12%), or were living abroad or had been lost to follow-up (13%) were not invited. The Ryff well-being scales were included in a postal questionnaire at age 52 sent to women who had completed at least one Women’s Health Questionnaire in the previous 2 years. Ethical approval for the study was obtained from the Greater Manchester Local Research Ethics Committee and the Scotland A Research Ethics Committee. Written, informed consent was obtained from the study member for each wave of data collection.

### Mastery

At age 52, women completed a 42-item version of the Ryff well-being scales. This included seven items capturing environmental mastery. Both positively worded (e.g., “I am good at juggling my time so that I can fit everything in that needs to get done”) and negatively worded (e.g., “I have difficulty arranging my life in a way that is satisfying to me”) items were included. Eligible responses were in six ordered categories ranging from *disagree strongly* to *agree strongly*. See Table 1 for the items used in the current study.

**Table 1.** Standardized Factor Loadings From Single-Factor and Two-Factor Measurement Models of Ryff’s Environmental Mastery Scale Included in the MRC National Survey of Health and Development at Age 52.

Single-factor measurement model		Two-factor measurement model	
Environmental mastery	Factor loading^a^	Environmental mastery skills	Factor loading^a^
I am quite good at managing the many responsibilities of my daily life	0.73	I am quite good at managing the many responsibilities of my daily life	0.80
I generally do a good job of taking care of my personal finances and affairs	0.60	I generally do a good job of taking care of my personal finances and affairs	0.64
I am good at juggling my time so that I can fit everything in that needs to be done	0.60	I am good at juggling my time so that I can fit everything in that needs to be done	0.65
I have been able to build a home and a lifestyle for myself that is much to my liking	0.70	Environmental mastery accomplishments	
*I often feel overwhelmed by my responsibilities*	0.55	I have been able to build a home and a lifestyle for myself that is much to my liking	0.75
*I have difficulty arranging my life in a way that is satisfying to me*	0.68	*I often feel overwhelmed by my responsibilities*	0.58
		*I have difficulty arranging my life in a way that is satisfying to me*	0.74
Goodness-of-fit statistics			
RMSEA	0.168		0.085
CFI	0.865		0.970
TLI	0.882		0.970
Chi-squared (*df*)	274.53(8)		66.26(7)

*Note*: RMSEA = root mean square error of approximation; CFI = comparative fit index; TLI = Tucker–Lewis index. Italicized items were reverse coded before analysis so high scores indicate high well-being; Standardized.

### Education

Prospectively collected information on study members’ educational qualifications achieved by age 26 years were grouped into *no qualifications*, *lower secondary* (‘O’-levels or equivalent, usually attained at 16 years), *advanced secondary* (‘A’-levels or equivalent, usually attained at 18 years), and *degree level or equivalent*.

### Occupational, Financial, Family and Health Characteristics, Childhood Circumstances, and Goals

Cumulative attachment to paid work was derived from employment status at ages 26, 36, 43, and 52. At age 52, women rated their working life to date on a 5-point scale. Women were grouped into those who found it very rewarding (the reference category), those who found it somewhat rewarding, and those who did not find it rewarding or had not ever been in paid work. Financial, family, and health difficulties over the last 12 months were also reported at age 52. Financial difficulties were indicated if the study member had sometimes or often had to go without things they really needed in the last 12 months because of a shortage of money. Three separate items captured occurrence of serious difficulties (e.g., because of their health or behavior) with the spouse or partner, children, and parents/other relatives in the last 12 months. Perceived changes in physical health and changes in nervous and emotional state in the last 12 months were each captured by a single item with five possible responses. Women who reported that their health had got a lot or a little better or had not changed were used as the reference category and compared with those who reported that their health had got a little or a lot worse.

Goals were captured at age 43, when study members reported whether they felt they had more to achieve in the family or home sphere, and in the work sphere, both on a 3-point scale. Answers were dichotomized to *nothing more to achieve* versus *something* or *much more* to achieve.

Childhood social class was based on father’s occupation at age 4 (or ages 11 or 15 if this was missing) and grouped according to the Registrar General’s classification. Women with a father in a skilled manual occupation were used as the reference group, as levels of mastery were hypothesized to be lower among those with the highest and lowest levels of childhood socioeconomic disadvantage.

### Statistical Analysis

Following previous psychometric validation in this cohort, one item was dropped from measurement of mastery (Abbott et al., 2006). Previous analysis of these data suggested that the remaining six mastery items load onto two correlated factors (Deeg, personal communication, 2013). We tested this formally with confirmatory factor analysis. We undertook structural equation modeling to model the two mastery subscales as a function of educational attainment. In separate models, we included (a) tangible benefits (occupational benefits, avoidance of financial and family difficulties, and avoidance of poor health), (b) goals for home/family life and working life, and (c) childhood socioeconomic circumstances. Finally, we included all covariates in a multiply adjusted model. The two mastery subscales were examined in the same structural equation models but are presented separately. Goodness of fit was assessed with the root mean square error of approximation (with values closer to 0 indicating better fit and a value of <0.06 generally considered good fit), the comparative fit index (ranges from 0 to 1 with higher values indicating better fit and 0.95 a suggested cut point), and the Tucker–Lewis index (with values closer to 1 indicating better fit and 0.95 a suggested cut point).

A total of 1,228 women took part in the age 52 follow-up, of whom 1,184 provided data for at least five of the six items comprising environmental mastery and were included in the analyses presented here. Compared with the original cohort of NSHD women, those who completed the Ryff environmental mastery scale had higher educational attainment and more socioeconomically advantaged childhoods. Full information maximum likelihood models were used so that study members who did not have complete data on the covariates of interest could be included under the assumption that the data were missing at random.

## Results

A two-factor model provided a considerably better fit to the data than a single-factor model for mastery (Table 1). We refer to the two factors as mastery skills and mastery accomplishments; the interfactor correlation was .42 in the education-adjusted model. Seven percent of women had degree level or higher qualifications and 38% had no formal qualifications (Table 2). The majority (67%) had been in work for three or more of the four adult sweeps. A higher percentage of women with high education rated their working life as rewarding compared with less educated women. Financial difficulties were more commonly experienced by less educated women. Difficulties with spouse, children, and other relatives and experience of worsening health did not differ by educational level. Nine years prior to the assessment of mastery, 26% of the sample felt they had nothing more to achieve in their family/home life and 35% felt they had nothing more to achieve in their work life; both these percentages were higher among less compared with more educated women. Higher percentages of women with high compared with low educational attainment had fathers in professional, managerial/technical, and skilled nonmanual occupations.

**Table 2.** Characteristics of Women From the MRC National Survey of Health and Development Who Completed the Mastery Scale as Part of the Women’s Health Questionnaire at Age 52.^a^

	All women *N* (%)	Percentage of women with low education^b^	Percentage of women with high education	*p*^c^
Education at age 26				
None	430 (38)			
O-level or lower	317 (28)			
A-level or equivalent	297 (27)			
Degree of higher	76 (7)			
Adult circumstances				
Number of sweeps in paid work to age 52				<0.001
0	28 (3)	4	1	
1	93 (9)	10	7	
2	209 (21)	22	19	
3	394 (40)	41	38	
4	263 (27)	23	35	
Working life rated as rewarding				<0.001
Very rewarding	362 (32)	27	43	
Somewhat rewarding	551 (48)	48	45	
Not rewarding/never in paid work	235 (20)	25	11	
Financial difficulties in last 12 months at age 52				0.001
Sometimes/often	226 (19)	22	14	
No	956 (81)	78	86	
Serious difficulties with partner/spouse				Yes vs. No 0.2
Yes	173 (15)	16	12	
No	902 (76)	77	76	
No partner/spouse	102 (9)	7	12	
Serious difficulties with any children				Yes vs. No 0.08
Yes	130 (11)	10	13	
No	905 (77)	79	72	
No children	143 (12)	11	15	
Serious difficulties with parent or other relative				0.9
Yes	336 (29)	28	29	
No	839 (71)	72	71	
Physical health change in last 12 months at age 52				0.3
Better/no change	893 (76)	75	77	
Worse	282 (24)	25	23	
Nervous and emotional state change in last 12 months at age 52				0.8
Better/no change	927 (79)	79	78	
Worse	248 (21)	21	22	
Goals				
Ambitions for family/home life at age 43				<0.001
Nothing more to achieve	283 (26)	31	16	
Something/much more to achieve	811 (74)	69	84	
Ambitions for working life at age 43				<0.001
Nothing more to achieve	375 (35)	41	22	
Something/much more to achieve	692 (65)	59	78	
Childhood circumstances				
Father’s social class				<0.001
I (Professional)	82 (7)	2	18	
II (Managerial and technical)	208 (19)	15	26	
IIINM (Skilled nonmanual)	241 (22)	17	29	
IIIM (Skilled manual)	317 (28)	35	15	
IV (Partly skilled)	220 (20)	25	10	
V (Unskilled)	53 (5)	7	1	

a Numbers are based on maximum available sample for each covariate. ^b^Low education defined as O-level or lower qualifications (typically obtained at age 16); ^c^Test for difference by educational level based on chi-squared test.

In the unadjusted model, women with low educational attainment had higher mastery-skills scores (Table 3, Model 1). Mastery-skills scores were lower among women who did not find working life rewarding, who experienced financial difficulties, and who had experienced declines in emotional health. The association between low education and greater mastery skills was larger in magnitude once these tangible benefits and stressors were controlled (Table 3, Model 2). Holding limited goals was not associated with mastery skills. Mastery skills were lower for women with fathers in the highest occupational classes compared with those with fathers in skilled manual occupations. The association between low education and higher mastery was attenuated by 23% (with the standardized coefficient dropping from 0.158 to 0.122) on adjustment for father’s social class (Table 3, Model 4).

**Table 3.** Relationship of Midlife Mastery Skills to Education Based on *n* = 1,184 Women in the MRC National Survey of Health and Development.

	Model 1: Education only	Model 2: Tangible benefits	Model 3: Goals	Model 4: Childhood circumstances	Model 5: Fully adjusted
Low education at age 26	0.158*	0.203*	0.142*	0.122*	0.150*
Sweeps in paid work to age 52		−0.042			−0.038
Working life not rewarding/no job		−0.147*			−0.156*
Working life somewhat rewarding		−0.087*			−0.081*
Financial difficulties at age 52		−0.193*			−0.188*
Serious difficulties with spouse		−0.019			−0.021
Serious difficulties with child		−0.005			0.000
Serious difficulties with parent/relative		−0.024			−0.030
Physical health decline		−0.011			−0.022
Nervous/emotional decline		−0.129*			−0.127*
Nothing more to achieve family/home life			0.046		0.046
Nothing more to achieve working life			0.047		0.046
Father’s social class (IIIM = reference)					
I (Professional)/II (Managerial/technical)				−0.150*	−0.151*
IIINM (Skilled nonmanual)				−0.051	−0.059
IV (Partly skilled)/V (Unskilled)				−0.074	−0.059
Goodness-of-fit statistics					
RMSEA	0.061	0.055	0.053	0.044	0.034
CFI	0.974	0.980	0.976	0.977	0.984
TLI	0.974	0.970	0.974	0.969	0.961

*Note*. RMSEA = root mean square error of approximation; CFI = comparative fit index; TLI = Tucker–Lewis index. Estimates are standardized.

* *p* < .05.

Educational attainment was not associated with mastery-accomplishment scores in the unadjusted model (Table 4, Model 1). Mastery-accomplishment scores were lower for women who did not find working life rewarding, those who experienced financial or family difficulties, and those who had declining emotional health. Controlling for these tangible benefits and stressors, an inverse association between educational attainment and mastery accomplishments was seen. Holding a more limited set of goals in the family/home was associated with higher mastery accomplishments and adjustment for goals partly attenuated the education–mastery—accomplishments association (Table 4, Model 3). We note that work accomplishment is not explicitly included in Ryff’s environmental mastery scale, whereas reference to accomplishments at home is. Women with fathers in the highest and lowest status occupations had lower levels of mastery accomplishments than those with fathers in skilled manual occupations and controlling for father’s social class explained some of the association between lower education and higher mastery accomplishments (Table 4, Model 4).

**Table 4.** Relationship of Midlife Mastery Accomplishments to Education Based on *n* = 1,184 Women in the MRC National Survey of Health and Development.

	Model 1: Education only	Model 2: Tangible benefits	Model 3: Goals	Model 4: Childhood circumstances	Model 5: Fully adjusted
Low education at age 26	0.063	0.130*	0.040	0.038	0.066
Sweeps in paid work to age 52		−0.057			−0.053
Working life not rewarding/no job		−0.255*			−0.269*
Working life somewhat rewarding		−0.133*			−0.127*
Financial difficulties at age 52		−0.223*			−0.217*
Serious difficulties with spouse		−0.139*			−0.142*
Serious difficulties with child		−0.112*			−0.104*
Serious difficulties with parent/relative		−0.086*			−0.092*
Physical health decline		0.009			−0.005
Nervous/emotional decline		−0.210*			−0.209*
Nothing more to achieve family/home life			0.110*		0.112*
Nothing more to achieve working life			0.034		0.036
Father’s social class (IIIM = reference)					
I (Professional)/II (Managerial/technical)				−0.252*	−0.147*
IIINM (Skilled nonmanual)				−0.161*	−0.097*
IV (Partly skilled)/V (Unskilled)				−0.131*	−0.059
Goodness-of-fit statistics					
RMSEA	0.061	0.055	0.053	0.050	0.049
CFI	0.974	0.980	0.976	0.989	0.979
TLI	0.974	0.970	0.974	0.985	0.961

*Note*. RMSEA = root mean square error of approximation; CFI = comparative fit index; TLI = Tucker–Lewis index. Estimates are standardized.

* *p* < .05.

## Discussion

Women with low educational attainment were found to have higher mastery skills than women with higher levels of education. Women with higher education tended to experience better occupational benefits and fewer financial stressors and, conditional on these factors (but not in models that were not adjusted for these factors), women with low educational attainment also had higher mastery accomplishments than women with higher levels of education. Both types of mastery were lower among women in households headed by a father in higher and lower status occupations and father’s social class contributed to explaining some of the inverse association between mastery and education. Several previous studies have shown that parental socioeconomic disadvantage (indicated by lower parental education or lower occupational social class) is associated with lower general sense of control in adult offspring (Krause, 1993; Mirowsky & Ross, 2007). However, one previous study found that social disadvantage is associated with higher levels of mastery (Ryff et al., 2003). We speculate that women in households headed by a father in middle and lower status occupations may have had more exposure to challenges in childhood and beyond and may have more frequently been in situations requiring them to develop skills to overcome challenges relative to women from the most advantaged families. Our finding of a non-linear association between father’s social class and mastery, with mastery being highest among those with fathers in skilled manual occupations, aligns with suggestions that early circumstances which are challenging but not overwhelming may facilitate the development of mastery (Luthar et al., 2000; Pearlin et al., 2007).

Our study may not have found a protective association with education because greater educational attainment of women in this cohort did not translate into increased opportunities to develop mastery. Although more compared with less educated women were more likely to report that their working life had been very or somewhat rewarding, we did not assess whether more educated women in this cohort had more leadership experience or autonomy in the workplace. However, we know that the norms for women in this birth cohort were for early marriage and childbearing with more highly educated women delaying childbearing for only a small number of years compared with later cohorts (Neuburger, Kuh, & Joshi, 2011). We were not able to assess the contribution of work–family conflict and role blurring in this cohort and it is possible that failure to account for these factors suppressed a positive association between education and mastery in this analysis. Recent work has shown that these are more commonly experienced by those with more qualifications (who are more likely to work in highly skilled occupations and have high job pressure) and that lack of time or energy to fully engage with family (work) due to work (family) has been associated with lower sense of mastery (Schieman & Narisada, 2014). Notably, our analysis showed that more highly educated women in this cohort did not have fewer problems with their spouse, children, or other relatives. Previous analysis of this cohort found that women occupying multiple roles of mother, wife, and paid work (more common among those with higher education) had the highest levels of health and well-being but did not differ in their levels of work and family stress compared with women occupying fewer roles (McMunn, Bartley, & Kuh, 2006).

Consideration of the measures used is warranted in interpreting these findings. While we used Ryff’s environmental mastery scale, the majority of studies used Pearlin’s mastery scale or scales capturing personal sense of control. Although there is some overlap at face value, the former accents skills to manage one’s time and affairs, whereas the scale developed by Pearlin accents the perception that one can influence current and future circumstances and personal sense of control accents the belief that outcomes depend upon actions. Although originally formulated as a single scale (Ryff, 1989), confirmatory factor analysis in the current study supported the hypothesis that items capturing skills to manage multiple demands and to juggle one’s time loaded onto a separate subscale from those capturing mastery over one’s circumstances. It is plausible that more educated women juggling a greater number of roles would have more challenges to overcome in successfully managing their multiple demands and this may explain the current findings and an earlier finding in this cohort that more highly educated women have more psychological distress symptoms (Hatch et al., 2007). If this is the case, then more highly educated women may plausibly not feel they have the skills to master their environment.

Mastery skills and mastery accomplishments were lower for those who had experienced recent financial difficulties, those who did not view their working life as rewarding, and those with worsening emotional state. These adult occupational, financial, and health factors were more common among those with lower education and therefore their inclusion in the regression models did not attenuate the relationship between higher education and lower mastery in the current study. As expected, having fewer goals for family/home life was associated with higher mastery-accomplishment scores. It may be helpful here to consider different processes or different stages of psychological control. Rothbaum et al. (1982) refers to a two-process model of perceived control: primary and secondary. High scores on the environmental mastery subscales may be achieved through a high perceived ability to directly control one’s environment or roles (i.e., primary control). Alternatively, they may be achieved through altering one’s goals and values to bring them in line with the environment rather than directly altering one’s environment (i.e., secondary control). Heckhausen et al. (Heckhausen & Schulz, 1993; Heckhausen et al., 2010) refer to this as compensatory secondary control. A related concept is that of goal readjustment. Readjustment of goals has been proposed as an adaptive strategy that helps people maintain emotional well-being in the face of unattainable goals (Tomasik & Silbereisen, 2014; Wrosch et al., 2003), although whether it is appropriate to label this psychological reaction to loss of primary control as a form of control is debated (Skinner, 1996). It has been hypothesized that secondary control may be a strategy more commonly employed when facing a chronic inability to obtain desired goals, as may be the case for those with low education (Seeman, 2008). Conceptually, readjustment of goals may help explain why women with lower levels of education may score more highly than less educated women on both mastery subscales. Although we did not have data allowing us to distinguish primary and secondary control, we explored women’s ambitions in the family/home and work spheres as one manifestation of this adjustment process. We hypothesized that low education would be related to relatively low ambitions. This hypothesis was supported, with women having a low level of education being less likely to say they have much more to achieve, and these differences in ambitions partly explained the relatively low levels of mastery accomplishments of the women with higher education level. While we hypothesized that lower ambition would be associated with greater mastery, Krause (1993) hypothesized that lower ambition would lead to greater financial problems in later life and hence lower mastery. Our results indicated that ambitions and financial problems were essentially independently related to mastery.

This study is based on a large, representative sample of British women in the 1940s and, unlike many previous studies, uses prospective data capturing circumstances in childhood and earlier adulthood. Some limitations of the study should be noted. First, although the measurement of own education preceded that of mastery, the latter was assessed only once and it is plausible that high levels of mastery result in successes in the educational sphere (Flouri, 2006). In fact, intervention studies indicate a reciprocal association between own education attainment and mastery (Skinner, Zimmer-Gemback, & Connell, 1998), but this was not assessed here. Second, Ryff’s psychological well-being scales, including the mastery scale, have been widely used although uncertainties over their psychometric properties remain (Abbott et al., 2006). Although statistical analysis supported the existence of two rather than one mastery scale within these data, it remains unclear how these factors should be labeled and whether this structure is replicated in other studies. The mastery-accomplishments factor includes two items that appear to more closely resemble Pearlin’s concept of mastery (i.e., the item on having difficulty in arranging life in a way that is satisfying and the item relating to status attainment having been able to build a satisfying home and lifestyle). However, it also includes an item on feeling overwhelmed which is less conceptually distinct from the mastery-skills factor.

Generalizability of findings from this cohort is a third important issue requiring consideration. If educational approaches and opportunities arising from education that have a bearing on mastery are different in subsequent cohorts or in cohorts outside of Britain, then we may find a different relationship between mastery and education in other studies, as noted earlier. Additionally, the current study is limited to women and there may be gender differences in the association between mastery and work (Ross & Wright, 1998) or family circumstances. Attrition was greater among women from lower parental education households, but the differences were fairly small in magnitude (66% of the analytical sample vs. 71% of the original sample having a parent with low education) and so we consider it unlikely that differential dropout among low education women with low mastery could explain the effect sizes seen here. Finally, we may have omitted variables that are important to understand the association between education and mastery. In particular, we were unable to empirically test possible suppression by work–family conflict using these data.

In conclusion, higher educational attainment was associated with lower levels of mastery skills in this sample of postwar baby boomer women in midlife. Those from the most and least advantaged childhood socioeconomic circumstances had the lowest levels of mastery and controlling for childhood circumstances attenuated some of the inverse association between education and mastery. This study highlights the long-standing contribution of childhood factors to a key component of psychological well-being and ageing well. It also suggests that education does not universally promote mastery and suggests that the education–mastery association is contingent upon context. However, women’s access to further education and its implications for career trajectories have changed substantially since the birth of this cohort and so this warrants further investigation in other cohorts.
